# The Effect of Light on the Germination of *Raphanus sativus* Seeds and the Use of Sprout Extracts in the Development of a Dermatocosmetic Gel

**DOI:** 10.3390/gels11070515

**Published:** 2025-07-02

**Authors:** Mihaela Carmen Eremia, Ramona Daniela Pavaloiu, Oana Livadariu, Anca Daniela Raiciu, Fawzia Sha’at, Corina Bubueanu, Dana Maria Miu

**Affiliations:** 1 National Institute for Chemical-Pharmaceutical Research & Development—ICCF, 112 Vitan Avenue, 3rd District, 031299 Bucharest, Romania; mihaelaceremia@yahoo.com (M.C.E.); pavaloiu.daniella@gmail.com (R.D.P.); fawzya.shaat@gmail.com (F.S.); corina.bubueanu@yahoo.com (C.B.); dana.miu92@gmail.com (D.M.M.); 2 Faculty of Biotechnologies, University of Agronomic Sciences and Veterinary Medicine of Bucharest, 59 Bd. Marasti, 011464 Bucharest, Romania; oana.livadariu@biotehnologii.usamv.ro; 3 Faculty of Pharmacy, Department of Pharmacognosy, University of Titu Maiorescu, Phytochemistry, Phytotherapy, 16 Gh. Sincai Street, 040441 Bucharest, Romania

**Keywords:** *Raphanus sativus* L. seeds, germination, sprout extracts, polyphenols, gels

## Abstract

This study investigates the influence of different light sources (sunlight, green, red, and white LED) on the germination of *Raphanus sativus* L. sprouts and the potential use of their sprout extracts in the development of natural dermatocosmetic gels. The bioactive fractions were extracted using simple methods and analyzed for total polyphenol content and antioxidant activity. Statistical analysis of weight, total phenolic content, and antioxidant activity of *Raphanus sativus* L. sprouts was performed using ANOVA. Sprouts exposed to green LED light showed the highest biomass (16.13 ± 0.38 g), while red LED light resulted in the highest total polyphenol content (3.28 ± 0.03 mg GAE/g fresh weight). The highest antioxidant activity (6.60 ± 0.08 mM Trolox/g fresh weight) was obtained under white LED. Although variations were observed, ANOVA analysis revealed that only sprout weight differed significantly among treatments (*p* < 0.001), while differences in polyphenol content and antioxidant activity were not statistically significant (*p* > 0.05). The extract with the highest antioxidant activity was incorporated as an active ingredient into Carbopol-based hydrogel formulations containing natural gelling agents and gentle preservatives. The resulting gels demonstrated favorable pH (4.85–5.05), texture, and stability. The results indicate that the light spectrum influences the germination process and the initial development of seedlings. Moreover, radish sprout extracts, rich in bioactive compounds, show promise for dermatocosmetic applications due to their antioxidant, soothing, and antimicrobial properties. This study supports the use of natural resources in the development of care products, in line with current trends in green cosmetics.

## 1. Introduction

Radish (*Raphanus sativus* L.), a member of the Brassicaceae family, is widely consumed in Romania and valued for its nutritional and medicinal properties. Its sprouts, in particular, have gained significant attention due to their high concentration of bioactive phytochemicals such as glucosinolates, isothiocyanates (e.g., sulforaphene), flavonoids, and various phenolic acids. These compounds contribute to the sprouts’ antioxidant, antibacterial, and anti-inflammatory effects, which are beneficial for dermatological and cosmetic formulations [1,2,3].

The polyphenolic profile of *R. sativus* sprouts—rich in rutin, gallic acid, and catechin—is a key source of antioxidant activity, which plays a crucial role in combating oxidative stress caused by environmental factors such as UV radiation and pollution. This mechanism is central to the prevention of skin aging, hyperpigmentation, inflammation, and carcinogenesis, as these compounds neutralize reactive oxygen species (ROS) and inhibit lipid peroxidation in skin cells [4,5]. Additionally, hydrolysis products of glucosinolates, such as isothiocyanates, activate the Nrf2-ARE signaling pathway, leading to the induction of phase II detoxifying enzymes and enhanced cellular protection against environmental stressors [6].

Topical application of *R. sativus* sprout extract through hydrogel systems not only supports the functional stability of these active constituents but also enhances skin hydration, a critical component for maintaining epidermal barrier integrity. These properties align well with the growing consumer demand for sustainable, biocompatible, and plant-based ingredients in green cosmetics [7]. Thus, incorporating *R. sativus* sprout extract into dermatocosmetic gels represents a targeted strategy for managing oxidative stress-related skin conditions while promoting skin regeneration, antimicrobial protection, and soothing effects.

Many studies have examined the functions of *R. sativus* in detoxification, digestive health, and as an antibacterial and anti-inflammatory agent throughout the last ten years [8,9,10,11,12]. The phytochemical content and biological activity of *R. sativus* vary significantly with plant part, developmental stage, and environmental conditions. Studies have confirmed that sprouts contain higher levels of antioxidants and phytochemicals than seeds or roots [13,14]. For instance, radish shoots may contain 3.8× more glucosinolates, 8.2× more isothiocyanates, and 6.9× more phenolics than roots [15].

Recently, light-emitting diode (LED) technology has been employed in controlled cultivation to stimulate the production of valuable secondary metabolites in sprouts due to its energy efficiency and spectral precision [16,17].

Studies have demonstrated that radish sprouts grown under specific LED wavelengths (e.g., red or blue light) show elevated levels of total phenolics, glucosinolates, and antioxidant activity, suggesting a strong influence of light spectrum on phytochemical development. Chen et al. (2023) analyzed radish sprouts cultivated under red and blue LED light for their chemical composition, as well as for their biological activities, such as antioxidant capacity and tyrosinase inhibition [18]. The findings revealed that red LED exposure promotes the accumulation of glucoraphasatin, enhancing the antioxidant potential of the sprouts, while blue LED exposure more effectively stimulates the production of compounds with tyrosinase inhibitory activity, suggesting a differential impact of light spectrum on bioactive compound synthesis.

Despite the increasing interest in LED-induced phytochemical enhancement, few studies have evaluated how different light spectra affect *R. sativus* sprout bioactivity for dermatocosmetic applications. We hypothesize that controlled light exposure can modulate the antioxidant potential of radish sprouts and that extracts from optimal treatments can be formulated into stable dermatocosmetic gels.

Based on this hypothesis, the present study investigates the effect of various light treatments—sunlight, green, red, and white LED—on the germination and phytochemical profile of *R. sativus* sprouts. The extract with the highest antioxidant activity was selected and incorporated into Carbopol-based hydrogel formulations, which were subsequently assessed for stability, pH, texture, and spreadability. This work aims to bridge the gap between controlled sprout cultivation and the development of effective plant-based skincare products, supporting innovation in green dermatocosmetic formulations.

## 2. Results and Discussion

### 2.1. The Effect of Light on the Development of R. sativus

**Figure 1:**
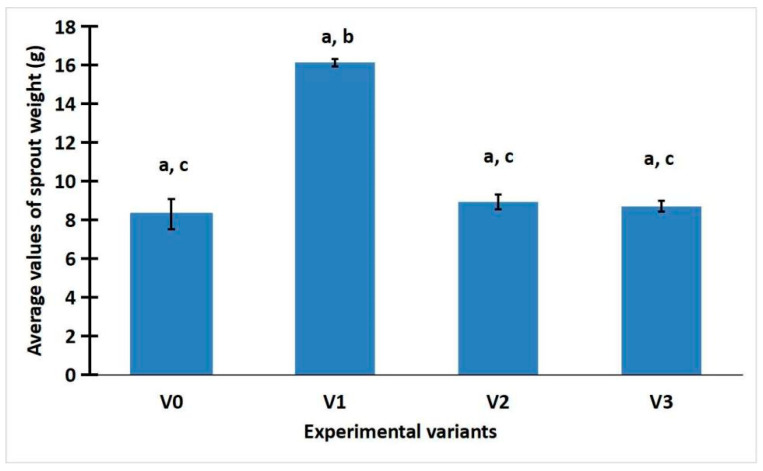
*R. sativus* sprout weight values. Data were expressed as mean ± SD. a. Highly significant difference between the LED-treated samples and the control group exposed to sunlight (*p* < 0.001, F > Fcrit), shown by ANOVA test. b. Significant differences between treatments V0/V1, V1/V2, and V1/V3, revealed by LSD test. c. Differences between treatments V0/V2, V0/V3, and V2/V3 are not significant, revealed by LSD test.

Looking at Figure 1, it can be seen that variant V1 (with green LED) reached higher values than the other variants. The average value of the weight of the gemoderivatives is 16.13 ± 0.38 g, compared to the average values of 8.93 ± 0.75 and 8.71 ± 0.57 g of experiments V2 with red LED and V3 with white LED, respectively, comparable to the control sample V0 (8.31 ± 1.56 g). When we examine how this experiment influenced the weight of *R. sativus* buds, it can be seen that the green light treatment plays a role in the amount of buds developed.

### 2.2. The Polyphenol Content and Antioxidant Activity

**Figure 2:**
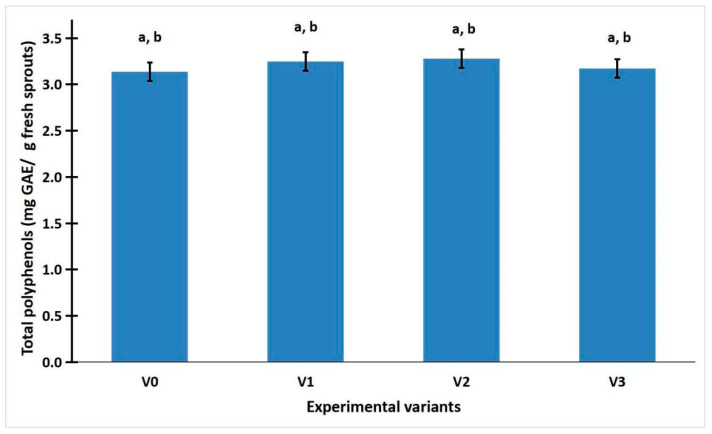
Total phenolic content in *R. sativus* sprouts. Data were expressed as mean ± SD. a. No significant difference between the LED-treated samples and the control group exposed to sunlight (*p* > 0.05, F < Fcrit), shown by ANOVA test. b. No significant differences between any treatment pairs, revealed by LSD test.

Figure 2 shows that polyphenols accumulated at a higher concentration in radish sprouts grown under red LED light (average value of 3.28 ± 0.03 of sample V2) compared to green LED light (average value of 3.25 ± 0.16 of sample V1), white LED light (average value of 3.17 ± 0.11 of sample V3), or control (average value of 3.14 ± 0.14 of sample V0).

The results are interesting in that the amount of sprouts obtained does not determine a higher concentration of polyphenols. Light is the determinant for their increased concentration.

**Figure 3:**
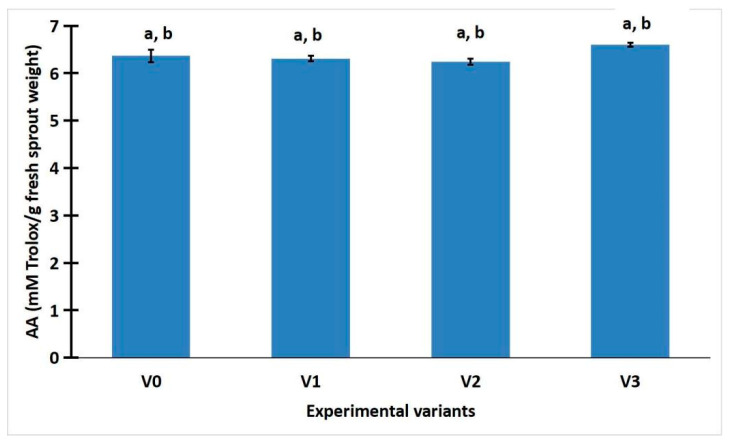
Antioxidant activity of *R. sativus* sprouts. Data were expressed as mean ± SD. a. No significant difference between the LED-treated samples and the control group exposed to sunlight (*p* > 0.05, F < Fcrit), shown by ANOVA test. b. No significant differences between any treatment pairs, revealed by LSD test.

Figure 3 shows that white LED light (average value of 6.60 ± 0.08 of sample V3) had higher antioxidant activity than the control LED light (average value of 6.36 ± 0.26 of sample V0), green (average value of 6.3 ± 0.12 of sample V1), and red light (average value of 6.25 ± 0.13 of sample V2).

The antioxidant properties of sprouted radishes were significantly enhanced by the white LED light treatment.

### 2.3. Statistic Analysis of Weight, Total Phenolic Content, and Antioxidant Activity of R. sativus Sprouts

ANOVA analysis was performed on the weight, total phenolic content, and antioxidant activity of *R. sativus* sprouts. The analysis of sprout weight revealed a highly significant statistical difference (*p* < 0.001) between the LED-treated samples and the control group exposed to sunlight (Supplementary Table S1). This finding rejects the null hypothesis and is further supported by the comparison of F and Fcrit values, where F exceeded Fcrit, indicating a statistically significant effect of LED illumination on sprout weight. The one-way ANOVA applied to total phenolic content and antioxidant activity under different lighting conditions (sunlight, white LED, green LED, and red LED) showed no statistically significant differences (*p* > 0.05), despite observable variations in mean values. The *p*-values ranged from 0.501 to 0.11, and in all cases, the F values were below the critical threshold (F < Fcrit), indicating that the null hypothesis could not be rejected. These results suggest that LED illumination does not have a statistically significant impact on the polyphenol content or antioxidant activity of *R. sativus* sprouts compared to natural light.

The LSD test was used to determine the statistical significance of differences between experimental variants. An outcome was considered “Significant” if the absolute difference between groups’ means exceeded the LSD threshold; otherwise, it was labeled “Not Significant”. Tables S2–S4 from the Supplementary Data summarize the results of the Least Significant Difference (LSD) test conducted on the weight, total phenolic content, and antioxidant activity of *R. sativus* sprouts. Following the ANOVA analysis, the LSD test identified statistically significant differences between certain treatment groups. Specifically, significant differences in sprout weight were observed between the following variant pairs: V0/V1, V1/V2, and V1/V3 (Supplementary Table S2). For all other comparisons across the measured parameters, no statistically significant differences were detected (Supplementary Tables S2–S4).

### 2.4. Characteristics of Dermatocosmetic Products Containing R. sativus Sprouts Extract

**Figure 4:**
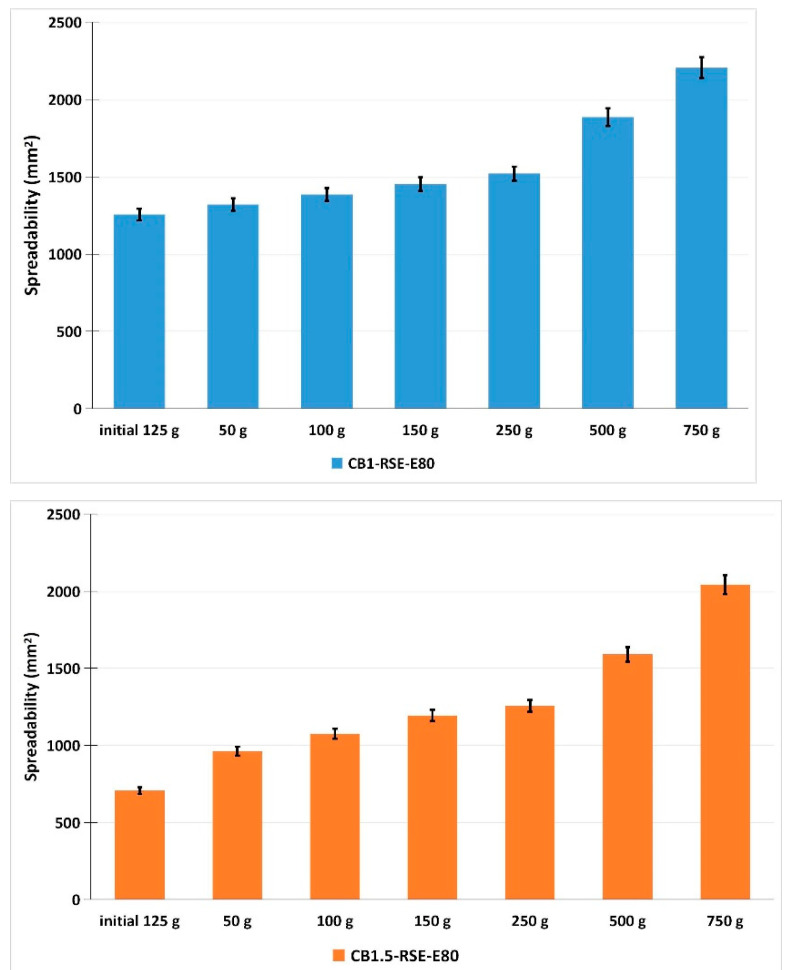
Spreadability profiles of dermatocosmetic gel formulations CB1-RSE-E80 (1% Carbopol) and CB1.5-RSE-E80 (1.5% Carbopol) containing *R. sativus* sprouts extract. The stretching surface area (mm^2^) was measured after each load application, demonstrating the spreadability behavior of the two formulations. Error bars represent standard deviations (n = 3).

*R. sativus* sprouts are a rich source of bioactive compounds, which contribute to their strong antioxidant properties, making radish sprout extract a good candidate for dermatocosmetic applications aimed at skin protection, rejuvenation, and barrier support. In Table 1, the composition of the formulations containing *R. sativus* sprouts extract is presented. The extract incorporated had a total polyphenol content of 3.16 mg GAE/g fresh weight sprouts and an antioxidant activity of 6.59 mM Trolox/g fresh weight sprouts. The dermatocosmetic gels were characterized through a series of analyses, including visual inspection, pH determination, spreadability testing, and texture profiling. The results are presented in Table 2 and Figure 4.

**Figure 5:**
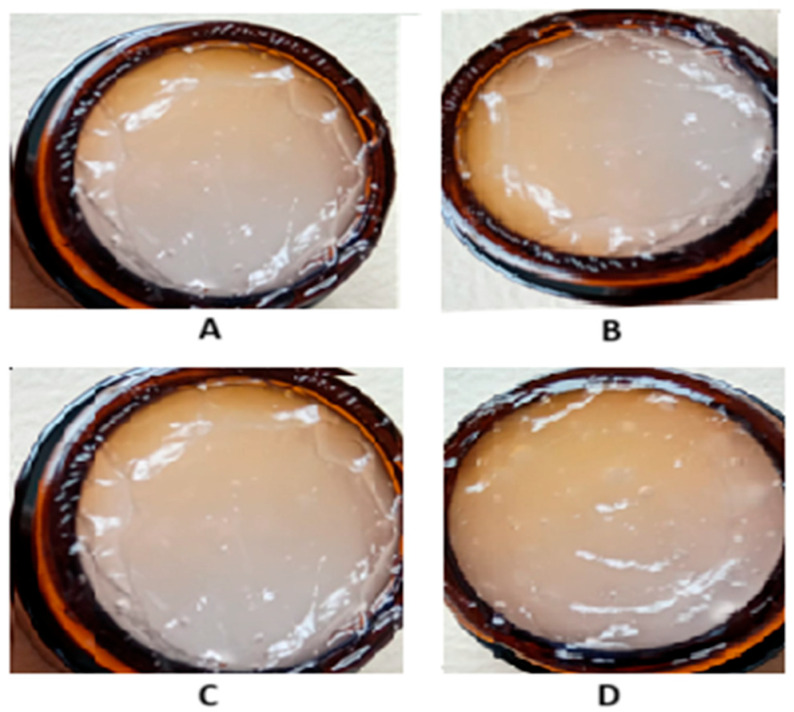
Macroscopic appearance of dermatocosmetic gel formulations containing *R. sativus* sprout extract immediately after preparation ((**A**)—CB1-RSE-E80 and (**B**)—CB1.5-RSE-E80) and after 30 days of storage at room temperature ((**C**)—CB1-RSE-E80 and (**D**)—CB1.5-RSE-E80).

From a macroscopic standpoint, all formulations demonstrated a high degree of uniformity, indicating consistent dispersion of active ingredients throughout each product. To support these observations, representative photographs of both gel formulations (CB1-RSE-E80 and CB1.5-RSE-E80) were captured immediately after preparation and after a 30-day storage period at room temperature. These are presented in Figure 5. The images reveal no signs of phase separation, sedimentation, or color/texture alteration, thereby confirming the physical stability and resilience of the formulations under standard storage conditions. Furthermore, statistical analysis showed that there were no significant differences in the pH values of the two formulations after 24 h and after 30 days of storage (*p* > 0.05). This indicates that the pH stability of the gels was maintained regardless of Carbopol concentration. The pH levels of these formulations also align well with the skin’s natural pH range, reducing the likelihood of irritation and supporting their suitability for extended topical use. Maintaining a pH between 4.5 and 5.5 is essential in dermatocosmetic products to protect the skin barrier, preserve the native microbiota, and avoid adverse reactions. Deviations from this range, particularly toward alkalinity (pH > 7), may deplete natural oils and compromise the skin’s protective function, while highly acidic products (pH < 3) can provoke irritation, especially on sensitive or compromised skin [19]. According to the Ojeda–Arboussa method, the spreadability analysis (Figure 5) confirmed that all formulations exhibit favorable application properties, further enhancing their appeal for dermal use. Regarding the texture, the formulation containing 1.5% Carbopol (CB1.5-RSE-E80) is firmer, more cohesive, and slightly more elastic than the 1% Carbopol gel. These improvements are in line with what is typically expected when increasing the concentration of Carbopol, a common thickening agent. The texture characteristics suggest that both formulations are well-structured and balanced.

### 2.5. Antioxidant Activity of the Gel Formulations

The antioxidant activity of the gel formulations was determined using the DPPH radical scavenging assay, with results expressed as both percentage inhibition and Trolox equivalent antioxidant capacity (mM Trolox/g gel). The formulation CB1-RSE-E80 exhibited the highest antioxidant activity, demonstrating 86.76% inhibition of DPPH radicals, corresponding to 1.646 mM Trolox/g gel. Similarly, the CB1.5-RSE-E80 formulation showed a slightly lower but still substantial antioxidant capacity, with 84.36% inhibition and 1.599 mM Trolox/g gel.

These findings confirm that the phenolic compounds retained their radical scavenging capacity after incorporation into the hydrogel matrix. The high inhibition values for both formulations indicate effective preservation of antioxidant properties, suggesting their potential utility in topical applications aimed at combating oxidative stress.

### 2.6. Discussions

This study demonstrates a significant effect of light spectrum on the biomass accumulation of *R. sativus* L. sprouts. Green LED light (V1) induced the highest sprout weight (16.13 ± 0.38 g), significantly outperforming red and white LED treatments, as well as the control. ANOVA and LSD tests confirmed these differences to be statistically significant (*p* < 0.001), supporting the hypothesis that green wavelengths can promote photosynthetic activity in lower levels, leading to enhanced biomass accumulation. These findings align with previous research indicating that light quality modulates growth dynamics in *Brassicaceae* crops and sprouts [20,21]. Such technologies enable precise manipulation of plant morphology and growth rates, offering major advantages for urban agriculture and microgreen production [22].

Despite the increased biomass under green LED, red LED (V2) yielded the highest total polyphenol content (3.28 ± 0.03 mg GAE/g fresh weight), suggesting that light-driven growth does not necessarily correlate with secondary metabolite biosynthesis. Although not statistically significant (*p* > 0.05), the trend is supported by previous research showing red wavelengths can enhance the accumulation of phenolics and flavonoids in various sprouting vegetables, including broccoli and kale [23]. Therefore, the red LED lighting may be strategically used to boost the nutraceutical quality of radish sprouts without necessarily increasing biomass.

Polyphenols, major antioxidant constituents of radish, have been well documented for their radical scavenging, anti-inflammatory, and even anti-carcinogenic activities [1,2,7,24,25]. The variation among LED treatments suggests that the biosynthesis of these compounds is influenced more by specific light signaling pathways than by biomass development alone, a notion corroborated by Chen et al. and Choi et al., who observed cultivar- and light-specific effects on phytochemical accumulation in radish sprouts [17,18]. Interestingly, white LED (V3) treatment resulted in the highest antioxidant activity, exceeding that of red and green LED-treated sprouts, though differences were not statistically significant. This observation may be attributed to the broader spectrum of white light stimulating a wider array of bioactive compounds, beyond just polyphenols [14,15].

The activation of particular plant photoreceptors and their downstream signaling pathways can help to explain the differences in responses seen under different LED light treatments. Phytochromes and cryptochromes, which control gene expression linked to plant development and stress adaptation, are known to be activated by red and blue light, respectively. For example, phenylpropanoid biosynthesis, a crucial process for the production of polyphenols, is anticipated to be upregulated when red LED light (660 nm) stimulates phytochrome B [22,23,26,27]. This process is in line with our finding that, although not displaying maximal biomass accumulation, sprouts exposed to red LED light had the highest total phenolic content.

The wide spectral range (400–700 nm) of white LED light, on the other hand, may excite several photoreceptors at once and increase the generation of ROS (reactive oxygen species). ROS serve as crucial secondary messengers in plants at low-to-moderate concentrations, inducing the production of enzymes such as peroxidases, polyphenol biosynthesis enzymes, and superoxide dismutase as well as antioxidant defense mechanisms [17,19,22,28,29]. Therefore, a ROS-mediated signaling cascade that increases the synthesis of flavonoids and phenolic acids may be the cause of the maximum antioxidant activity shown in sprouts treated with white LEDs. Interestingly, the NADPH oxidase–MAPK–NAC transcription factor pathway, which is triggered by oxidative stress brought on by light exposure, is frequently used to mediate this process [30,31,32].

According to these molecular pathways, photoreceptor-regulated transcription and oxidative signaling are two ways that LED light influences biomass production as well as the qualitative profile of bioactive substances. Thus, modifying LED wavelength and intensity during sprout development is a potent way to customize phytochemical composition for particular uses, including dermatocosmetic formulations rich in antioxidants [33,34].

One limitation of the current study is the exclusive focus on seedling weight as the primary growth parameter, without accompanying morphological assessments such as seedling length, leaf number, or root development. These parameters are critical for understanding the full physiological impact of different LED treatments on plant morphology [34,35]. Their inclusion would have provided a more comprehensive evaluation of growth responses beyond biomass accumulation. Future studies will aim to incorporate a broader set of morphological characteristics to enable a more detailed assessment of light-induced effects on early-stage plant development.

It should be mentioned as a limitation as well that the DPPH assay, which is extensively accepted and informative, was the only method used to measure antioxidant activity in this work. This assay only provides a partial view of the antioxidant profile. Future research will incorporate additional assays like ABTS and CUPRAC to have a more thorough understanding of the antioxidant potential of radish sprout extracts. These methods will assist in capturing a broader spectrum of antioxidant mechanisms in both lipid and aqueous environments, such as electron transfer and radical scavenging.

Similar to our results, Goyeneche et al. and Kajszczak et al. noted that antioxidant capacity in *R. sativus* leaves and sprouts can be highly variable and dependent on both the extraction method and physiological stage [8,12]. These findings underscore the complexity of phytochemical interactions and the need for more targeted investigations into the specific metabolites contributing to antioxidant potential.

The integration of *R. sativus* sprout extracts into topical gel formulations revealed strong dermatocosmetic potential. Both formulations (CB1-RSE-E80 and CB1.5-RSE-E80) showed good physical stability, uniform texture, and pH levels within the physiologically acceptable range of 4.5–5.5. This pH range is critical for maintaining skin barrier integrity and minimizing irritation, particularly for sensitive or compromised skin [19]. *R. sativus* extracts have been previously associated with antioxidant, antimicrobial, and anti-inflammatory properties, making them suitable candidates for skin protection and rejuvenation products [4,10,11].

In addition to the physical and pH stability evaluations, the antioxidant activity of the final gel formulations was quantitatively assessed using the DPPH radical scavenging assay to ensure that the functional properties of the incorporated radish sprout extract were retained post-formulation. The results demonstrated high levels of radical inhibition in both gels, with CB1-RSE-E80 exhibiting 86.76% inhibition (1.646 mM Trolox/g gel) and CB1.5-RSE-E80 showing 84.36% inhibition (1.599 mM Trolox/g gel). These findings confirm that the antioxidant compounds, primarily phenolic constituents, remained active after being integrated into the hydrogel matrix. This step is essential for validating the functional integrity of plant-based formulations, as the antioxidant performance of the extract must be preserved in the final product to justify its dermatocosmetic potential. Without this validation, claims regarding antioxidant efficacy would remain speculative. The data thus support the role of *R. sativus* sprout extract as a viable ingredient for formulations targeting oxidative stress-related skin concerns.

The pH values of the formulations remained stable and within the skin-compatible range throughout the storage period, with no statistically significant differences between the two formulations, confirming their suitability for topical use.

The improved firmness and cohesiveness observed with 1.5% Carbopol suggest better retention on the skin surface, which could enhance the delivery and efficacy of bioactive ingredients. Previous formulations with radish extracts have demonstrated promising results in wound healing and anti-aging contexts, further validating the use of such extracts in functional skincare products [36].

Therefore, the current formulations should be further evaluated to substantiate antioxidant efficacy, skin compatibility, and user acceptability. The use of *R. sativus* sprouts—especially when cultivated under optimized light conditions—represents a sustainable and effective approach to enhancing the bioactivity of dermatocosmetic products. To complement the current findings, additional structural parameters such as viscosity and water loss, along with detailed phytochemical profiling of the incorporated extract (e.g., by HPLC-MS), are currently under investigation and will be included in a future study.

## 3. Conclusions 

This study examined how different types of LED light influence the germination and bioactive properties of *R. sativus* sprouts, and explored their potential use in natural dermatocosmetic gels. Green LED light significantly increased sprout weight, while red and white LEDs led to higher levels of polyphenols and antioxidant activity, respectively. However, only the increase in sprout weight was statistically significant based on ANOVA results.

The extract with the highest antioxidant activity—obtained under white LED light—was used to develop gel formulations. These gels showed good physical stability, a skin-friendly pH, and preserved antioxidant capacity after incorporation. Together, the findings suggest that radish sprout extracts, especially when produced under specific light conditions, have potential as natural, bioactive ingredients in skincare products.

To ensure scientific transparency and strengthen the rigor of this study, several limitations should be acknowledged. First, the evaluation of antioxidant activity was based solely on the DPPH assay, which, while widely accepted, provides only a partial view of the antioxidant potential and does not encompass the full range of antioxidant mechanisms. Second, the extract used in the formulations was not subjected to detailed phytochemical profiling using advanced techniques such as HPLC-MS, limiting our ability to identify and quantify the specific bioactive compounds responsible for the observed effects. Third, the study did not include any in vitro biological testing—such as cell-based assays or skin models—to assess the bioavailability, cytotoxicity, or functional activity of the extracts or the final formulations. Lastly, the physicochemical characterization of the gels was limited to basic parameters like pH, texture, and spreadability. Important aspects such as viscosity, water loss, and product stability over extended storage conditions were not investigated in this phase of the research.

Future work will address these gaps by using additional antioxidant assays (such as ABTS or CUPRAC), performing full phytochemical profiling (e.g., HPLC-MS), and conducting in vitro biological testing. We also plan to explore the wound healing and anti-inflammatory potential of the formulations, along with a more in-depth evaluation of their physicochemical properties over time.

## 4. Materials and Methods

### 4.1. Materials and Reagents

Sodium carbonate, Folin–Ciocalteu reagent, gallic acid, Carbopol 940, sodium hydroxide, and glycerin were purchased from Sigma-Aldrich (Merck Group, Darmstadt, Germany). 2,2-diphenyl-1-picrylhydrazyl was purchased from Tokyo Chemical Industry (TCI) (Tokyo, Japan), and peppermint essential oil (*Mentha piperita* L.) from Fares, Romania. All solvents (methanol, ethanol) were acquired from Adra Chim SRL (Bucharest, Romania).

### 4.2. Experimental Design

The experiment aimed to evaluate the influence of different light spectra on the early development of *R. sativus* microplants (microgreens/sprouts). *R. sativus* seeds obtained from a commercial source were surface-sterilized, inoculated, and incubated under sterile conditions (microgreens/sprouts), following the protocol described by Raiciu et al., 2020b [37]. Seed inoculation was performed in transparent recipients on sterile gauze, moistened with 20 mL of sterile distilled water. The seeds were initially kept in the dark for 24 h to encourage uniform germination, after which they were transferred to a controlled growth environment with a daily photoperiod of 16 h.

The experimental plan included four experimental variants: natural sunlight (V0, control), green LED light (V1, 525 nm), red LED light (V2, 640 nm), and white LED light (V3, 470–640 nm). Each treatment was applied for 10 consecutive days. The technical specifications and spectral characteristics of the LEDs correspond to those reported by Raiciu et al., 2020b [37] for red and white LEDs, and by Livadariu et al., 2019 and Raiciu et al., 2020a for green LEDs [20,21]. Treatment allocation was randomized to minimize positional and environmental bias. Each light variant (V0–V3) was grown in three independent containers, and each container was considered one repetition. After growth, the fresh weight of sprouts was determined, as well as total polyphenol content and antioxidant capacity using methanolic extracts of sprouts. Methanolic extracts were prepared by extracting the entire microplants—including both aerial parts and radicles—in 100% methanol, using a fresh tissue-to-solvent ratio of 1:4 (*w*/*v*) for 4 days, followed by centrifugation at 8000 rpm. For each repetition (container), a random selection of sprouts was made to obtain a sufficient volume of extract for biochemical analyses. The 4-day extraction period was chosen based on preliminary optimization trials to maximize polyphenol yield while minimizing degradation. The resulting supernatants were used to determine total polyphenol content and antioxidant capacity.

Statistical analysis for weight, total phenolic content, and antioxidant activity of *R. sativus* sprouts was conducted using one-way ANOVA, and statistical significance was determined at *p* < 0.05. The Least Significant Difference (LSD) test was employed to identify meaningful differences between treatment groups for weight, total phenolic content, and antioxidant activity of *R. sativus* L. sprouts.

### 4.3. Determination of the Total Polyphenol Content of the R. sativus Sprouts Extracts

The total polyphenol content was determined using the Folin–Ciocalteu method [38]. In brief, 1 mL of the sample was mixed with 10 mL of distilled water and 1 mL of Folin–Ciocalteu reagent (previously diluted 1:10 *v*/*v*). The mixture was then brought to a final volume of 25 mL by adding a 5% (*w*/*v*) aqueous sodium carbonate solution. After incubation for 30 min, the absorbance was measured at 760 nm using a UV/VIS spectrophotometer (Jasco V-630, Portland, OR, USA). The total polyphenol content was calculated from a standard calibration curve of gallic acid in the concentration range of 0.01–0.1 mg/mL (y = 0.01322x + 0.0272; R^2^ = 0.99574). The results were expressed as milligrams of gallic acid equivalents per gram of fresh sprout weight (mg GAE/g).

### 4.4. Determination of the Antioxidant Activity of Radish Sprouts Extracts

The antioxidant activity of methanolic extracts of radish sprouts was measured using the Sanchez-Moreno et al. assay [39]. This spectrophotometric method is based on the decrease in the absorbance of the free radical DPPH• in the presence of antioxidants. Briefly, each sample (0.6 mL) was mixed with 2.4 mL of DPPH methanolic solution (concentration 0.025 g/L). The inhibition of the DPPH radical was measured spectrophotometrically using a UV/VIS spectrophotometer (Jasco V-630, Portland, OR, USA) at a wavelength of 546 nm after 30 min.

The antioxidant activity was calculated using Equation (1) and a calibration curve with Trolox as standard:

AA (%) = [(A_DPPH_ − A_Sample_)/A_DPPH_] × 100 (1)

where A_DPPH_ is the absorbance of the control solution and A_Sample_ is the absorbance of the reaction in the presence of DPPH solution.

The Trolox calibration curve was prepared by measuring the DPPH radical scavenging activity of Trolox solutions at concentrations ranging from 5 to 50 µM under the same experimental conditions. The Trolox calibration curve used for antioxidant quantification via the DPPH assay was y = 51.701x + 1.690, with a coefficient of determination R^2^ = 0.9983, indicating excellent linearity within the tested range. The antioxidant activity was expressed in mMTrolox/g fresh sprout weight.

### 4.5. Formulation of Dermatocosmetic Products Containing R. sativus Sprouts Extract

The extract from *R. sativus* sprouts cultivated on gauze and illuminated with white LED light (V3) was selected for product development due to its superior antioxidant activity. The methanolic extract was concentrated, and the resulting precipitate was re-suspended in 80% ethanol, yielding a solution suitable for gel incorporation, labeled RSE-E80. The ethanolic extract was analyzed for total polyphenol content and antioxidant activity using the methods described in Section 4.3 and Section 4.4.

Two hydrogel formulations containing *R. sativus* sprouts extract, designated CB1-RSE-E80 and CB1.5-RSE-E80, were prepared using two Carbopol 940 concentrations (1% and 1.5%). Carbopol was hydrated in purified water for at least 24 h, and glycerin (Merck, Darmstadt, Germany) was added as a dispersing agent. Neutralization was achieved using a 2% sodium hydroxide solution (Merck Group, Darmstadt, Germany), added under vigorous stirring to the hydrated polymer to form a stable gel matrix. The *R. sativus* extract and peppermint essential oil (*Mentha piperita* L.) (Fares, Romania) were then incorporated into this matrix under continuous stirring until full homogenization was achieved. Peppermint essential oil (0.3%) was added not only for its pleasant aroma but also for its multifunctional role in the formulation. It offers antimicrobial activity, contributes to a refreshing sensory experience, and imparts a gentle cooling effect on the skin, which helps enhance the gel’s overall soothing and calming properties.

The initial pH of the *R. sativus* sprouts extract (RSE-E80) was 5.55 ± 0.1. In comparison, the aqueous Carbopol 940 base (before neutralization) had a more acidic pH of 2.85 ± 0.1, typical of its carboxylic functionality. In order to reach a final gel pH appropriate for topical administration, these values were crucial in directing the neutralization stage utilizing sodium hydroxide.

### 4.6. Characterization of Dermatocosmetic Formulations

#### 4.6.1. Visual Inspection

The appearance was assessed by applying a thin layer of gel onto a microscope slide and examining it with a magnifying glass (4.5×). Texture, uniformity, color, and odor were noted.

#### 4.6.2. pH Measurement

The pH of each formulation was measured using a digital pH meter (Mettler Toledo, Columbus, OH, USA). Samples were thoroughly mixed before measurement. After a 30 s stabilization period, three measurements were taken from different areas of each sample. If variation exceeded ±0.2 pH units, the procedure was repeated.

#### 4.6.3. Spreadability Assessment

Spreadability was evaluated 24 h after formulation using the Ojeda–Arbussa method. One gram of the sample was placed between two glass plates for one minute. A series of increasing weights (50 g to 750 g) additional weights (50 g, 100 g, 150 g, 250 g, 500 g and 750 g) were applied in 1 min intervals, to achieve a total weight of 175 g, 225 g, 275 g, 325 g, 625, and 875 g, and the spread diameter was measured in millimeters. The spread surface area (Si) was calculated using the following formula:

Si = di^2^ (π/4) (2)

where

Si = spreading surface area (mm^2^) for the applied mass i (g);

di = mean diameter (mm) of the sample under load.

#### 4.6.4. Texture Analysis

The texture analysis was performed using a TX-700 Texture Analyzer (Lamy Rheology, Champagne-au-Mont-d’Or, France) equipped with a 10 kg load cell. A two-cycle compression test (Texture Profile Analysis, TPA) was conducted to simulate product application and evaluate the following: (*i*) firmness (hardness)—maximum force during the first compression, indicating resistance to deformation; (*ii*) cohesiveness—ratio of the area under the second compression curve to the first, reflecting structural integrity; and (*iii*) springiness—distance recovered between the two compressions.

A cylindrical sample holder filled with gel (30 g), ensuring a smooth, level surface, and the TPA analysis was performed using a ½ spherical probe. Test parameters were set as follows: compression speed—0.8 mm/s, compression distance—10 mm, trigger force—5 g (0.05 N), interval between cycles—5 s, and room temperature to simulate skin conditions. Each formulation was tested in triplicate to ensure result accuracy.

#### 4.6.5. Antioxidant Activity

The antioxidant activity of the gel formulations was assessed using the DPPH radical scavenging assay to verify the preservation of active phenolic compounds after formulation. This assay evaluates the hydrogels’ capacity to neutralize free radicals, serving as an indicator of their potential to mitigate oxidative stress upon topical application. Approximately 1 g of each gel was combined with 10 mL of methanol to extract the antioxidant constituents. The mixture was vortexed for 5 min and then filtered to obtain a clear extract. A 0.6 mL aliquot of the gel extract was mixed with 2.4 mL of a DPPH methanolic solution (0.025 g/L). The reaction mixtures were incubated in the dark at room temperature for 30 min to avoid light-induced degradation. The degree of DPPH radical inhibition was determined spectrophotometrically using a UV/VIS spectrophotometer (Jasco V-630, Portland, OR, USA) at a wavelength of 546 nm.

#### 4.6.6. Stability

Gel formulations were stored in amber glass recipients at room temperature (25 °C) for 30 days. Physical appearance, pH, and odor were monitored after 30 days.

### 4.7. Statistical Analysis

All the measurements were performed in triplicate; the resulting data were statistically analyzed and presented as mean values ± standard deviation (mean ± SD). Statistical analysis for weight, total phenolic content, and antioxidant activity of *R. sativus* sprouts was conducted using one-way ANOVA, and statistical significance was determined at *p* < 0.05. The Least Significant Difference (LSD) test was employed to identify meaningful differences between treatment groups for weight, total phenolic content, and antioxidant activity of *R. sativus* sprouts.

In addition to the analyses performed on sprout extracts, a statistical comparison of the pH values of gel formulations (CB1-RSE-E80 and CB1.5-RSE-E80) was conducted using an independent samples *t*-test. pH values were measured in triplicate and expressed as mean ± standard deviation. A *p*-value < 0.05 was considered statistically significant.

## Figures and Tables

**Table 1:** Dermatocosmetic gel formulations containing *R. sativus* sprouts extract.

Formulation Component	CB1.5-RSE-E80	CB1.5-RSE-E80
Carbopol 940	1%	1.5%
Glycerin	8%	8%
RSE-E80	2.5%	2.5%
Peppermint essential oil	0.3%	0.3%
Sodium hydroxide	q.s	q.s.
Purified water	Up to 100%	Up to 100%

**Table 2:** Characteristics of dermatocosmetic gel formulations containing *R. sativus* sprouts extract.

Characteristics	CB1-RSE-E80	CB1.5-RSE-E80
Organoleptic evaluation—after 24 h	Appearance: Homogeneous Color: White Smell: Aromatic, specific	Appearance: Homogeneous Color: White Smell: Aromatic, specific
pH—after 24 h	5.05 ± 0.03	4.85 ± 0.05
Organoleptic evaluation—after 30 days, room temperature	Appearance: Homogeneous Color: White Smell: Aromatic, specific No signs of phase separation, sedimentation, or color/texture alteration,	Appearance: Homogeneous Color: White Smell: Aromatic, specific No signs of phase separation, sedimentation, or color/texture alteration,
pH—after 30 days, room temperature	5.15 ± 0.04	4.95 ± 0.03
Texture	Firmness: 0.824 N Cohesiveness: 0.529 N Springiness: 0.681 mm	Firmness: 0.986 N Cohesiveness: 0.542 N Springiness: 0.737 mm

## Data Availability

The original contributions presented in this study are included in the article/Supplementary Material. Further inquiries can be directed to the corresponding author.

## Supplementary Materials

The following supporting information can be downloaded at: https://www.mdpi.com/article/10.3390/gels11070515/s1, Table S1: A one-way ANOVA test for the *Raphanus sativus* L. sprouts’ weight, total phenolic content and antioxidant activity; Table S2: The statistical significance determined by LSD test values of *Raphanus sativus* L. sprouts’ weight; Table S3: The statistical significance determined by LSD test values of *Raphanus sativus* L. sprouts’ total phenolic content; Table S4: The statistical significance determined by LSD test values of *Raphanus sativus* L. sprouts’ antioxidant activity.
