# Supplementary material for: The Effect of Light on the Germination of Raphanus sativus Seeds and the Use of Sprout Extracts in the Development of a Dermatocosmetic Gel
Source: Gels. 2025 Jul 2;11(7):515. doi: 10.3390/gels11070515 (PMC12294505; doi:10.3390/gels11070515)
Supplement: Supplementary file 1 [file gels-11-00515-s001.zip › gels-3701250-supplementary.pdf]

Table S1. A one-way ANOVA test for the *Raphanus sativus* L. sprouts' weight, total phenolic content and antioxidant activity.

ANOVA test	F	p-value	Fcritic
Weight	48.757	1.742 E-05	4.066
Total phenolic content	0.857	0.501	4.066
Antioxidant activity	2.775	0.110	4.066

Table S2. The statistical significance determined by LSD test values of *Raphanus sativus* L. sprouts' weight.

Comparison	Abs Difference	LSD Value	Significance
V0 vs V1	7.82	1.752	Significant
V0 vs V2	0.62		Not Significant
V0 vs V3	0.4		Not Significant
V1 vs V2	7.2		Significant
V1 vs V3	7.42		Significant
V2 vs V3	0.22		Not Significant

Table S3. The statistical significance determined by LSD test values of *Raphanus sativus* L. sprouts' total phenolic content.

Comparison	Abs Difference	LSD Value	Significance
V0 vs V1	0.11	0.229	Not Significant
V0 vs V2	0.14		Not Significant
V0 vs V3	0.03		Not Significant
V1 vs V2	0.03		Not Significant
V1 vs V3	0.08		Not Significant
V2 vs V3	0.11		Not Significant

Table S4. The statistical significance determined by LSD test values of *Raphanus sativus* L. sprouts' antioxidant activity.

Comparison	Abs Difference	LSD Value	Significance
V0 vs V1	0.05	1.752	Not Significant
V0 vs V2	0.11		Not Significant
V0 vs V3	0.24		Not Significant
V1 vs V2	0.06		Not Significant
V1 vs V3	0.29		Not Significant
V2 vs V3	0.35		Not Significant
